# Trans-sacral screw fixation of posterior pelvic ring injuries: review and expert opinion

**DOI:** 10.1186/s13037-022-00333-w

**Published:** 2022-07-27

**Authors:** Navid Ziran, Cory A. Collinge, Wade Smith, Joel M. Matta

**Affiliations:** 1 St. Joseph’s Hospital and Medical Center, 500 W. Thomas Road Suite 850, Phoenix, AZ 85013 USA; 2 Texas Health Harris Methodist Hospital Fort Worth, Orthopedic Specialty Associates, 800 Fifth Avenue, Suite 300, Fort Worth, TX 76104 USA; 3 Swedish Medical Center, 701 E. Hampden Ave. Suite 515, Englewood, CO 80113 USA; 4 The Steadman Clinic, 181 W. Meadow Dr. Suite 400, Vail, CO 81657 USA

**Keywords:** Iliosacral, Trans-sacral, Screw, Posterior pelvic ring, Pelvic fracture, Injury mechanism

## Abstract

Posterior pelvic ring injuries (i.e., sacro-iliac joint dislocations, fracture-dislocations, sacral fractures, pelvic non-unions/malunions) are challenging injury patterns which require a significant level of surgical training and technical expertise. The modality of surgical management depends on the specific injury patterns, including the specific bony fracture pattern, ilio-sacral joint involvement, and the soft tissue injury pattern. The workhorse for posterior pelvic ring stabilization has been cannulated iliosacral screws, however, trans-sacral screws may impart increased fixation strength. Depending on injury pattern and sacral anatomy, trans-sacral screws can potentially be more beneficial than iliosacral screws. In this article, the authors will briefly review pelvic mechanics and discuss their rationale for ilio-sacral and/or trans-sacral screw fixation.

## Introduction

Pelvic ring injuries can be life-threatening with substantial co-morbidity and mortality [1]. Sequelae of failed treatment include limb-length imbalance, sitting problems, gait abnormalities, disorders of bowel and bladder, sexual dysfunction, neurologic conditions, acute and chronic pain, nonunion and others [2]. Our biomechanical understanding of these injuries has improved substantially over the past few decades and has resulted in improved surgical treatments. A complete discussion of pelvic mechanics is beyond the scope of this article; however, we would like to review relevant mechanical points. Further, we will discuss our rationale for trans-sacral and ilio-sacral screw utilization for posterior pelvic ring pathologies.

### Structural stability of the pelvic ring

The pelvis is composed of three separate bones: the sacrum and the right and left innominate bones. The sacrum is attached to the innominate bones via the sacro-iliac (SI) joint and the strongest ligaments in the body: the anterior/posterior SI ligaments, the interosseous ligament, and the sacro-spinous and sacro-tuberous ligaments [3]. The anterior pelvic ring is connected by the interpubic ligaments and an elastic fibrocartilaginous disc. Numerous muscles attach to the pelvis including the glutei, adductors, rotators, abdominal, and paraspinal muscles. These muscles impart substantial forces on the pelvis and play a role in deformity and fixation failure after reparative surgery.

Chamberlain first described the clinical method to assess stability and mobility of the pubic symphysis and the SI joints in 1930 [4, 5]. The vertical mobility of the pubic symphysis is measured on a radiographic anteroposterior (AP) view with the patient in a flamingo stance (standing on one leg and the other non-weightbearing and hanging freely). Chamberlain set the following normal values for vertical motion (y-axis) of the pubic symphysis: adult male 0–0.5 mm, adult nulliparous female 0–1.0 mm, adult multiparous female 0–2.0 mm. All cases with symphyseal mobility > 2 mm had pain in the pelvic joints. In 1984, Walheim et al. [6] confirmed these results demonstrating the majority of translational motion of the pubic symphysis in all 3 planes was less than 2 mm; most of the translation was in the y-axis plane.

**Fig. 1:**
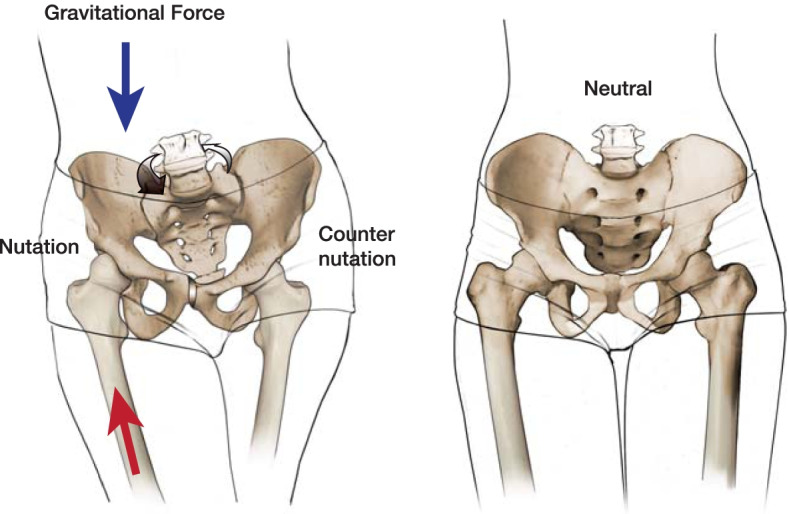
Illustration demonstration the effect of loading on the pelvis during gait. During weight-bearing the reactive normal force vector, directed cranially, results in nutation of the ipsilateral pelvis/sacrum. The sacral base (promontory) tilts anteriorly and inferiorly and the distal sacrum tilts posteriorly relative to the ilium (*nutation*, or “to nod”). The ipsilateral hemipelvis shifts slightly in the y-axis through the pubic symphysis. The opposite motion occurs in the contralateral pelvis (counternutation). The motion is exaggerated in the images for demonstration. This alternating motion during gait is reciprocal unilateral motion

During gait, the pubic symphysis moves in concert with the innominate bones and the sacrum. It has been demonstrated in healthy human subjects that the sacrum obliquely flexes and extends around a horizontal axis centered at the interosseous ligament of the SI joint [7]. This motion is called nutation and counternutation (Fig. 1). In Latin, nutation means “*to nod*” and, per Kapandji [8], it refers to the “anterior rotation of the sacrum about an axis constituted by the axial ligament so that the promontory moves inferiorly and anteriorly while the apex of the sacrum/tip of the coccyx move posteriorly.” Counternutation is defined as the opposite motion with the tip of the sacrum moving anteriorly. During gait, these are complementary motions as the loaded hemipelvis undergoes nutation during heel strike/stance phase while the contralateral side undergoes counternutation. This complementary motion is known as reciprocating unilateral motion and can be up to 2 degrees. There is considerable variation between humans due to differences in bony structure and ligamentous laxity (SI ligaments, interosseous SI ligament, sacro-spinous/sacro-tuberous ligaments). This nutation/counternutation motion can also be simultaneously bilateral. Bilateral nutation increases the pelvic outlet while counternutation increases the pelvic inlet. This nutation/counternutation motion about the SI joint is especially important during childbirth as demonstrated by Farabeuf at the beginning of the twentieth century [9]. In Farabeuf’s work, he showed that 90 degrees of hip flexion and internal rotation of the thighs increased the pelvic outlet while thigh external rotation increased the pelvic inlet. This flexibility in the posterior pelvic ring, in addition to the laxity in the pubic symphysis induced by pregnancy, facilitates passage of the fetus. There has been extensive literature published on the SI joint which is beyond the scope of this article. For a thorough review on the SI joint, the reader is encouraged to access the review by Vleeming et al. [10].

### Fixation modalities for posterior pelvic ring injuries

Nutation and counternutation relate to this discussion because they are unique to the SI joint and pelvis. The interdigitating grooves and ridges of the SI articular surface afford it the highest coefficient of friction than any diarthrodial joint and have been demonstrated to resist shearing [11–13]. Sacro-iliac fracture-dislocations may represent an intermediate fracture stable configuration. While there is no universally accepted standard of posterior pelvic ring fixation in North America, these fundamental concepts of pelvic mechanics contribute to our rationale for pelvic fixation.

The conventional work-horse surgical fixation treatments for posterior pelvic ring injuries include the ilio-sacral screw (ISS), posterior tension band plating, and anterior SI joint plating [14, 15]; lumbopelvic fixation [16] may also have a role but will not be included in this discussion. More recently, trans-sacral screws (TSSs) that span the entire posterior pelvis have been advocated as a tool for posterior pelvic fixation [17–19]; however, their specific indications have not been well-defined. These typically-long screws span the S1 or S2 body and capture the dense, cortical/subchondral bone of the contralateral SI joint and iliac cortex. The anterior pelvic ring contributes approximately 40% of pelvic ring stability [20]. Therefore, anterior pelvic ring fixation also contributes to the stability of posterior pelvic ring fixation however, this discussion will focus on posterior pelvic ring fixation methods.

Trans-sacral screws are placed down anatomical corridors that are rendered safe only if a patient’s anatomy and fracture alignment allow passage. Although much work has been done to anatomically define these corridors [21], the intraoperative safety of implant insertion rests with decision-making by the experienced pelvic/acetabular surgeon. These screws function, in most cases, by applying compression across the fracture or dislocation. This compression resists translational or rotational forces until healing is achieved. For some fracture pathologies, more secure fixation with more than one screw may be beneficial. It is well recognized that more points of fixation spread out in space, with preferential abutment against side-walls to resist translational forces, is optimal. Some injured pelvi are atypical or “dysmorphic-operatively,” and corridors for screw placement may be small or even non-existent [22]. These cases should be determined pre-operatively based on radiographic review of imaging and incorporated into the pre-operative plan. Interpretation of “safe” corridors is made even more difficult by existence of osteoporosis or imaging problems such as retained contrast media, bowel gas, and obesity [23].

The sacral ala (Latin meaning “wing”) is the upper surface of the lateral part of the first sacral vertebrae. It is lateral to the sacral promontory, triangular in shape, and articulates with the ilium. If fractures are isolated to the sacral ala and do not propagate vertically down the sacrum, we typically do not fix these injuries. There is no discontinuity within the axial skeleton since the normal force is still transmitted through an intact SI joint and the majority of the sacrum. Thus, for complete, unilateral sacral ala fractures, no fixation is needed. However, weight-bearing can be restricted if there is concern for fracture propagation.

If the sacral fracture line is vertical and complete, but minimally displaced, one trans-sacral screw (TSS) ± another TSS or an ISS is beneficial to prevent further displacement. Our anecdotal experience and that recently reported by others is that patients, especially the elderly, mobilize earlier and with less pain after fixation [24]. However, in elderly patients with limited pre-operative mobility status or morbid medical problems, conservative treatment may be more appropriate. For unsuccessful closed reductions or for displaced sacral fractures with reasonable posterior soft tissue, open reduction and internal fixation with table skeletal fixation is our preferred treatment [25]. Although early-career orthopaedic surgeons are performing more percutaneous fixation of the posterior pelvic ring and less open surgery, we believe that open surgery provides the best results [26]. The impact of this change from open to percutaneous fixation on open surgical volume and/or surgeon proficiency is unknown. For fixation of these displaced sacral injuries, we frequently utilize two or more ISSs or TSSs and consider a posterior tension band plate for fixation. If there is significant displacement, segmental comminution, and/or a horizontal fracture pattern, we consider the supplemental use of lumbopelvic fixation. Most displaced SI fracture-dislocations are also treated with open reduction (anterior or posterior) and fixation with posterior ilium screw fixation and 1 or more TSSs or ISSs. Sacro-iliac joint dislocations are best reduced posteriorly and “keyed-in” at the inferior aspect of the SI joint articulation. Due to the more inherent stability of the SI joint, 1 ISS screw may be satisfactory, however 2 are frequently used in patients with unstable injuries or poor bone stock. Bilateral sacral fractures are best fixed utilizing 2 or more TSSs from opposite directions. In patients with sacral dysmorphism, in which a trajectory across the entire sacrum is difficult, TSS placement may be difficult. For difficult sacral anatomies, we utilize one or two ISSs placed in either S1, S2, or both. In patients with SI joint dislocations, we utilize one ISS – either into S1 or S2. The compressed SI joint is inherently more resistant to vertical shear than sacral fractures. Lastly, pelvic malunions/nonunion pose a difficult pathology to address. These cases are addressed in stages (i.e., anterior–posterior-anterior) and fixed posteriorly with posterior tension band plating and 1 or more TSS. Fixation strategies for various disruptions of the pelvic ring are shown in Table 1.

**Table 1:** Preferred fixation choices for posterior pelvic ring injuries. For dysplastic sacra with recessed sacral ala, etc., the authors attempt an S1 screw but may also place an S2 screw

Unilateral sacral ala fracture, vertical, complete	No fixation
Unilateral, vertical, complete, minimally displaced	1 trans-sacral screw + 1 iliosacral screw/trans-sacral screw
Unilateral, vertical, complete sacral fracture, displaced	Posterior tension band plate + 1 trans-sacral screw
Sacro-iliac joint dislocation	1 iliosacral screw
Sacro-iliac fracture joint dislocation	Innominate bone reduction and fixation + 1 TSS
Bilateral, vertical sacral fracture	2 TSS from opposite directions
Pelvic malunion/nonunions	Posterior tension band plate + 1 or more TSS

Trans-sacral screws, in addition to posterior tension band plating, are well-suited for reconstruction of pelvic malunions and nonunions. Matta reported successful use of TSS in pelvic malunions/nonunions in addition to sacral fractures [27]. Gardner and Routt also reported successful utilization of TSS in a cohort of 56 patients with good early surgical results [19].

**Fig. 2:**
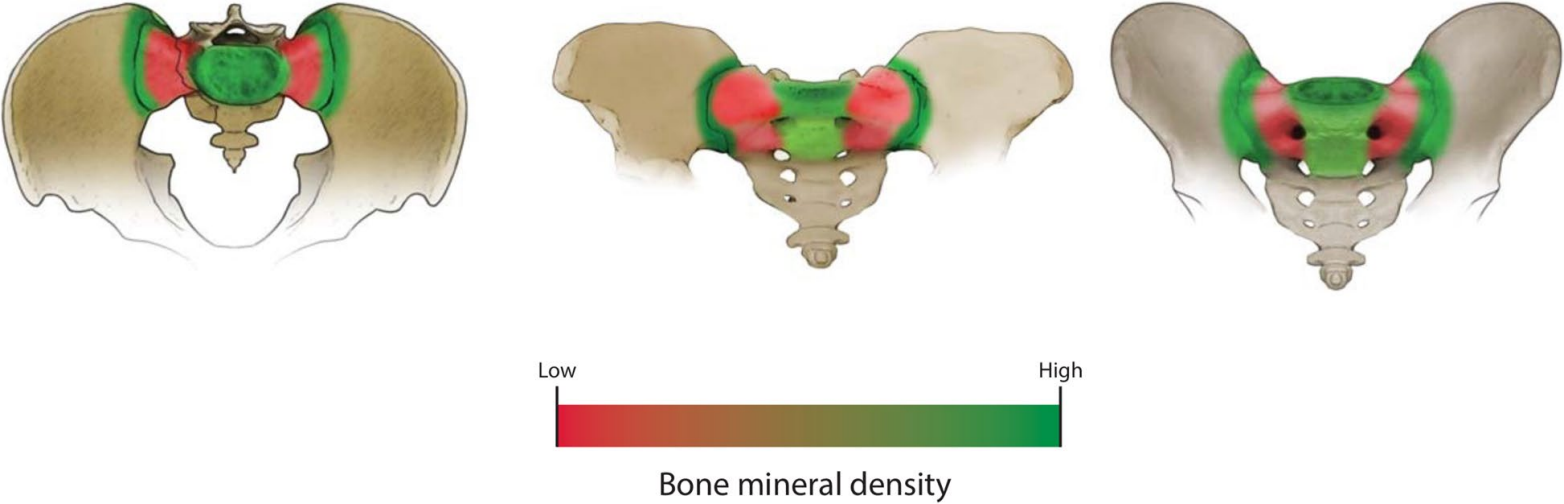
Illustration demonstrating the color-coded bone mineral density of the posterior pelvic ring. Higher bone density is located near the SI joints, the S1 body, and the superior endplate of S1

There is controversy over which screws (ISSs vs. TSSs) are most suitable biomechanically since bone quality is an important variable in ISS/TSS mechanics [28]. More recent studies have offered insight into the bone density of the sacrum. The regions with the highest bone density are near the SI joint, the S1 body, and near the superior endplate of S1 (Fig. 2) [29, 30]. Poor cancellous bone density is found in the sacral ala, especially in those individuals with osteoporosis [31]. More recent literature has also demonstrated better bone quality in S1 over S2 [31].

**Fig. 3:**
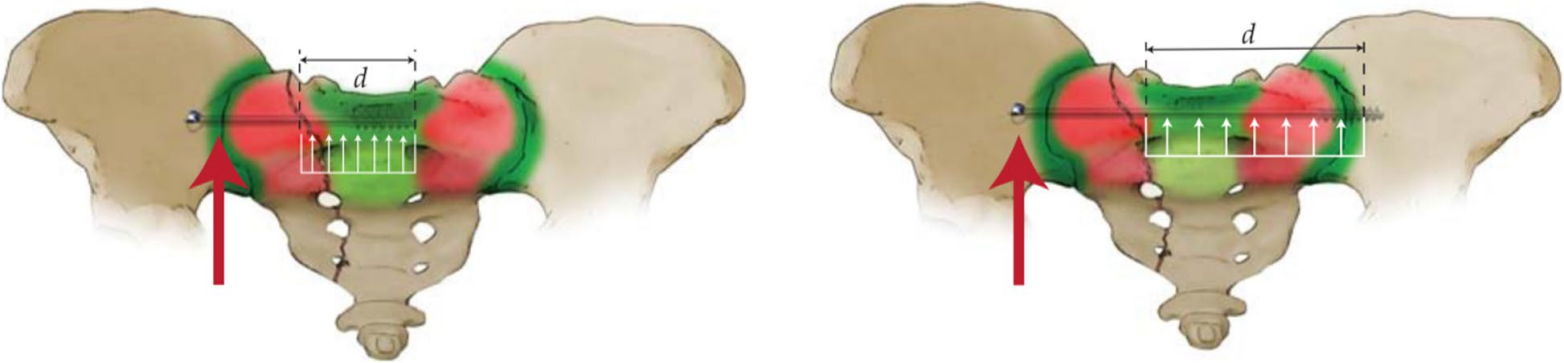
Illustration of a vertical sacral fracture with both iliosacral screw fixation and trans-sacral screw fixation. During loading on the ipsilateral side, the force vector is directed cranially. This force is distributed along a longer implant length in the trans-sacral screw versus the iliosacral screw. Further, the contralateral iliac cortex has higher bone density than the S1 body

After reduction and fixation of complete SI dislocations the main mode of failure is vertical shear. The contact area between the sacrum and the innominate bone resists this shear via friction. Compression of the SI joint via an ISS or TSS screw further increases resistance to an upward vertical shear force between the sacrum and the innominate bone. In osteoporotic individuals, however, the bone in the sacral body may be poor. Subsequent compression of the SI joint or sacral fracture may be lower in these cases; this compression also provides resistance to vertical shear forces. For these reasons, the authors prefer to place a TSS with screw threads that purchase the stronger bone of the contralateral iliac cortex. During loading, the normal force to the limb imparts a vertically directed force (Fig. 3, red arrow). The TSS is longer than the ISS, and the vertical shear forces are subsequently distributed along a longer length implant compared to an ISS (white arrows representing distance d, Fig. 3). The distance from the sacral fracture to the sacral body is significantly less than the distance from the sacral fracture to the contralateral iliac cortex. Zhao et al. confirmed these findings in their finite element analysis (FEA) comparison between ISSs and TSSs [32]. Therefore, the TSS is more resistant to cranial displacement because of its 1) distribution of cranial forces along it longer length, 2) greater screw thread purchase in the contralateral iliac cortex, and 3) potential increase in compressive forces of the fracture/SI joint. Because most of the purchase of either ISSs or TSSs occur either in the S1 body or the contralateral cortex, the authors utilize partially-threaded screws—as there does not appear to be any mechanical advantage to fully-threaded screws. Further, fully-threaded screws may impede compression of the fracture site.

**Fig. 4:**
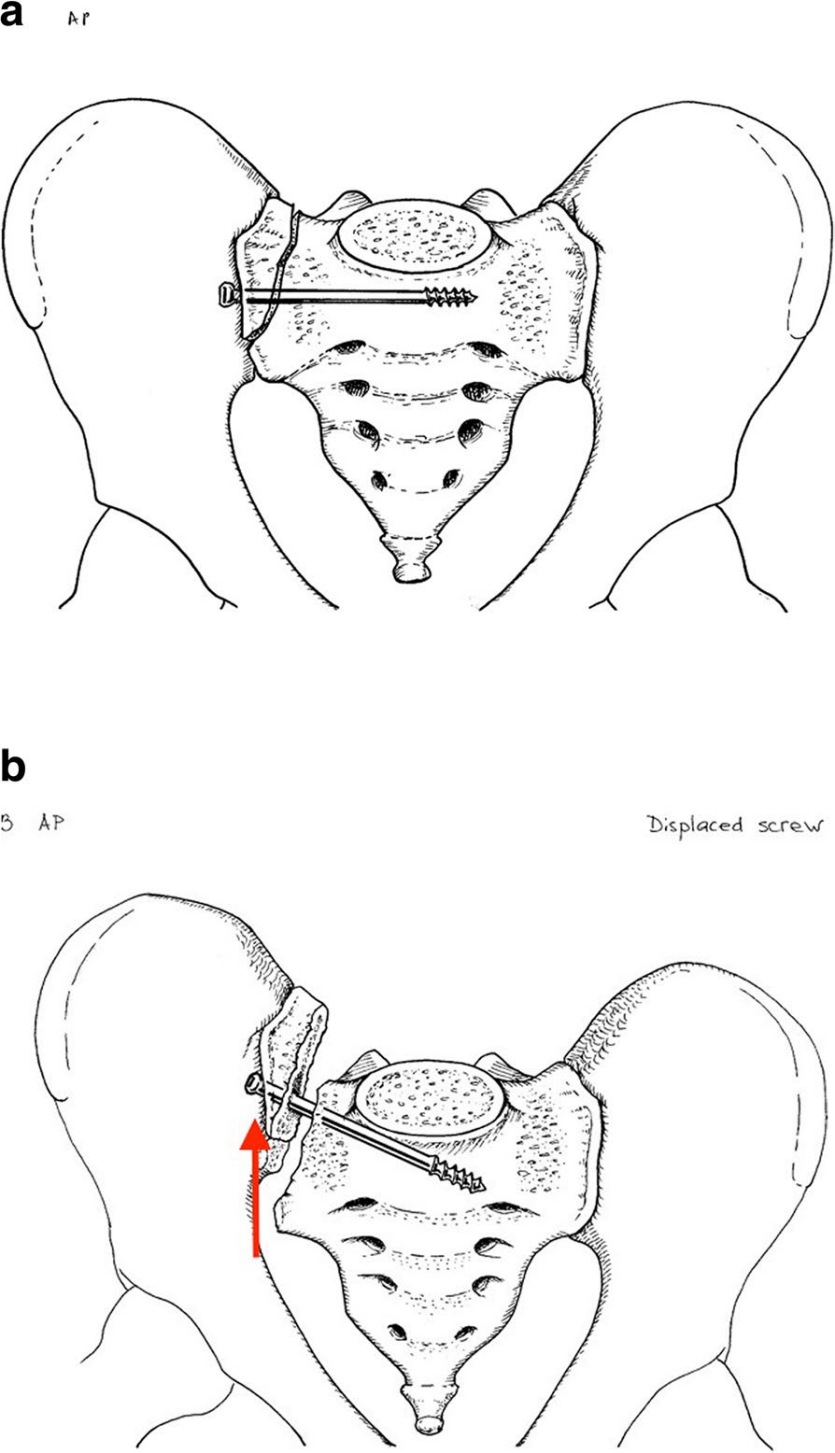
Drawing of a right SI fracture-dislocation treated with an iliosacral screw (**A**). The usual mode of failure for this screw is rotation in the coronal plane with subsequent cranial fracture displacement (**B**)

**Fig. 5:**
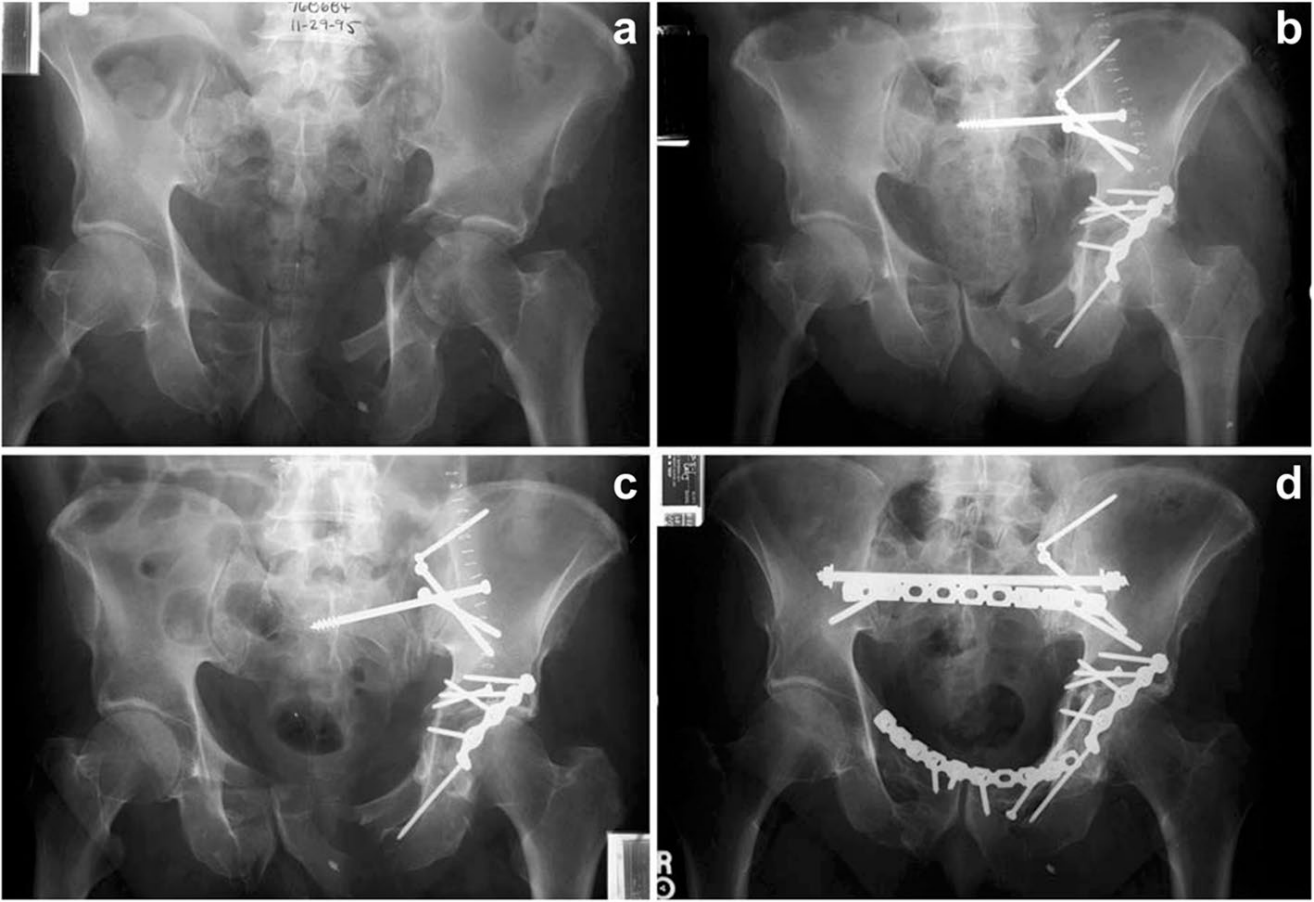
Radiographic images of a combined left SI fracture-dislocation and T-shaped acetabular fracture (**A**). After fracture fixation (**B**), the posterior pelvic fixation failed (**C**) necessitating revision with a trans-sacral screw, posterior tension band plate, and anterior ring fixation (**D**)

When ISSs do fail, the main mode of failure is a rotational moment causing 1) the screw to rotate within the cancellous bone of the ala or 2) implant failure. This moment causes cranial displacement of the affected hemipelvis with subsequent rotation of the IS screw relative to the vertical S1 coronal plane midline (Fig. 4). As noted above, this type of ISS failure is a prime indication for salvage using TSS fixation (Fig. 5). The reason for the re-displacement, in this particular example, is the lack of secure screw purchase in the sacrum and lack of anterior pelvic ring fixation. Elderly individuals with poor bone quality (osteopenic or osteoporotic) who frequently sustain these injuries, are not always compliant with weight-bearing, and without anterior ring fixation are vulnerable to displacement. Routt reported 13% ISS failure rate for sacral fractures [33]. Matta reported 11% ISS failure rate for pelvic malunions/nonunions [27]. Some authors have reported success with cement augmentation and ISS placement [34].

**Fig. 6:**
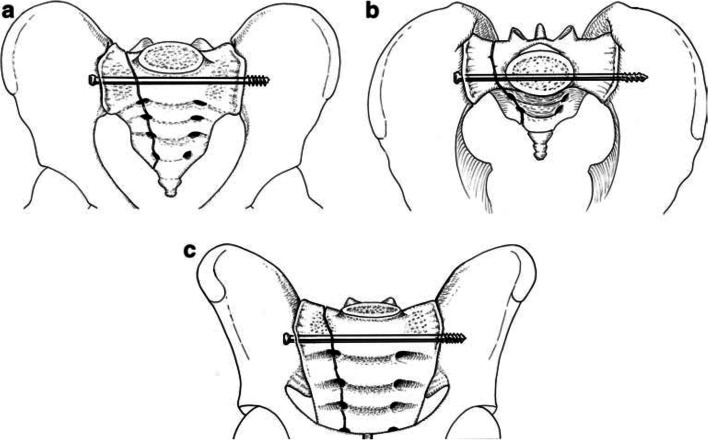
Antero-posterior (AP) pelvis, inlet, and outlet drawings of a right vertical sacral fracture fixed with a right trans-sacral screw

**Fig. 7:**
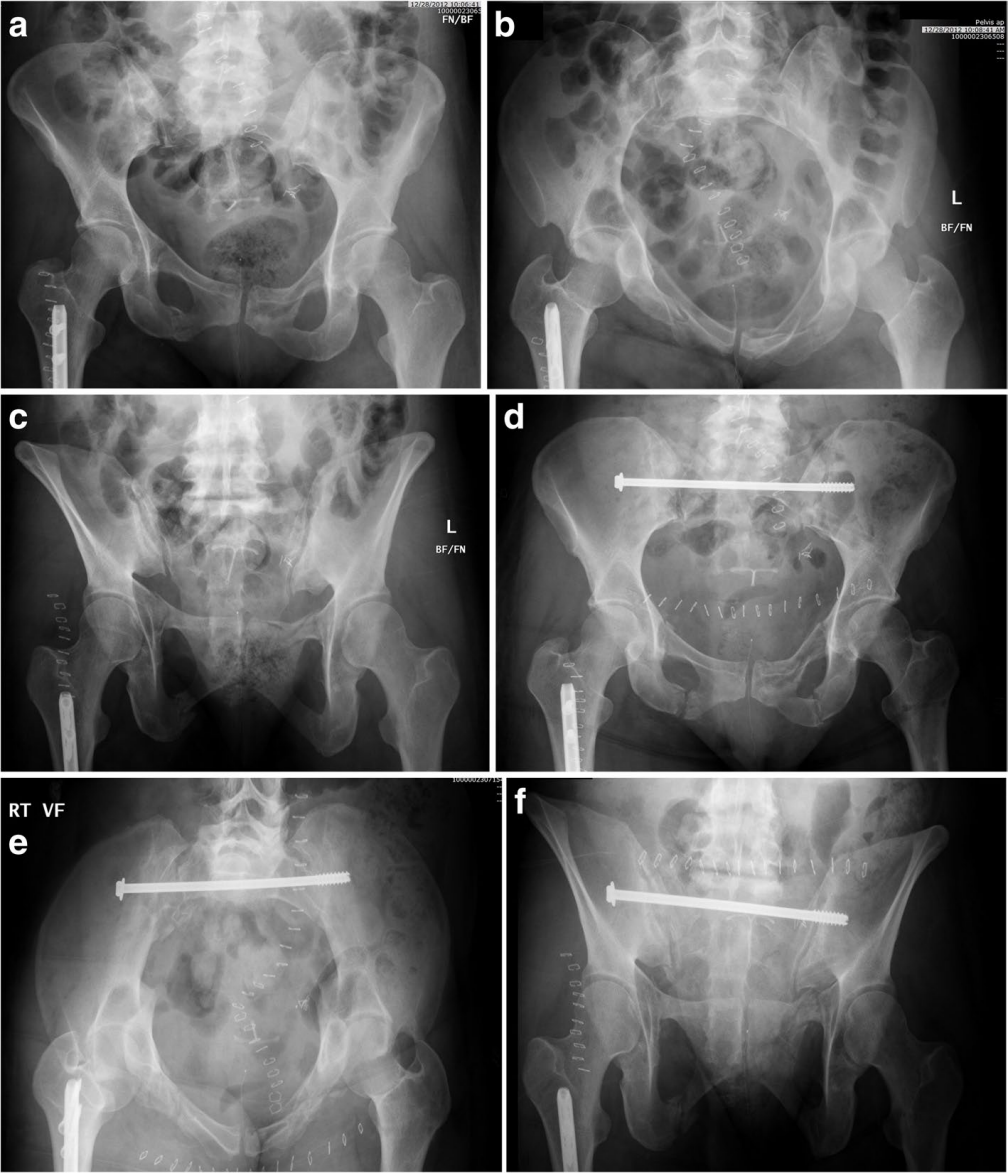
Radiographic pelvic images demonstrating a right SI fracture-dislocation (**A**-**C**) reduced and fixed with a trans-sacral screw (**E**-**F**)

**Fig. 8:**
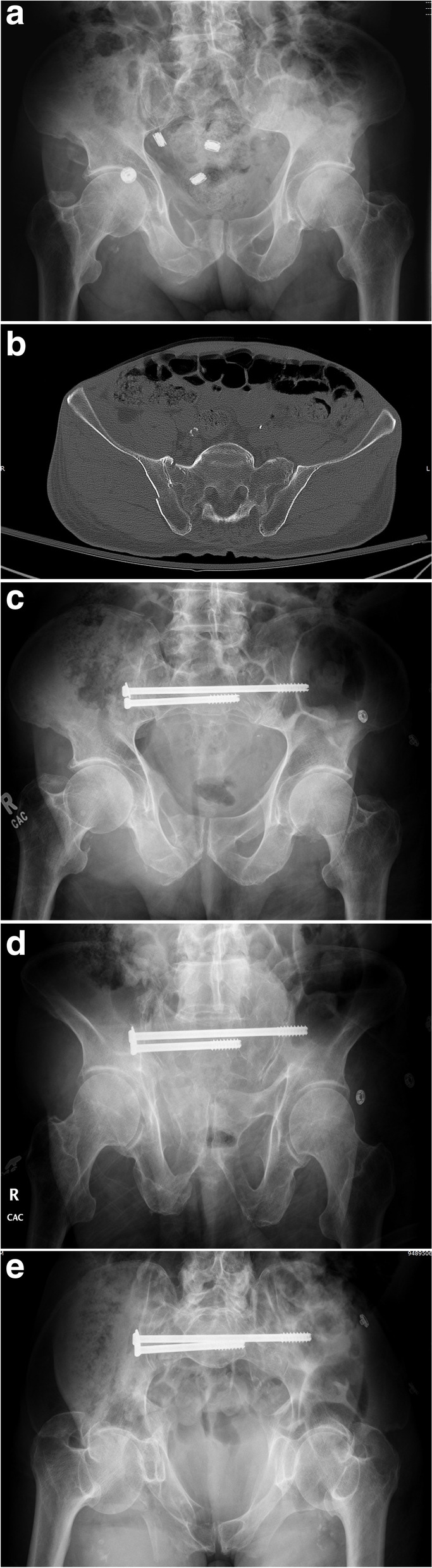
Radiographic and axial CT pelvic image of a right SI fracture-dislocation (**A**-**B**) treated with a right trans-sacral screw and right ilio-sacral screw to prevent implant failure and fracture re-displacement (**C**-**E**)

**Fig. 9:**
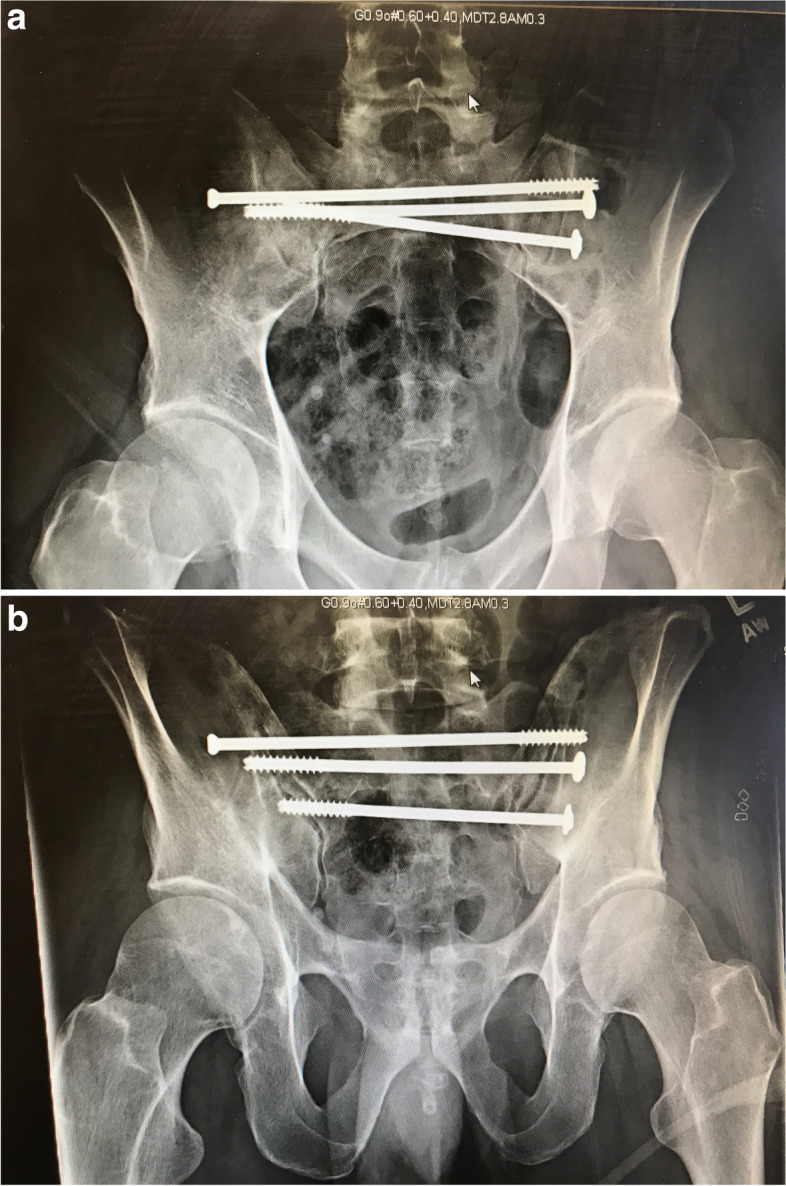
Post-operative pelvic radiographs (**A**-**B**) of a posterior pelvic ring injury fixed with three trans-sacral screws in both S1 and S2

**Fig. 10:**
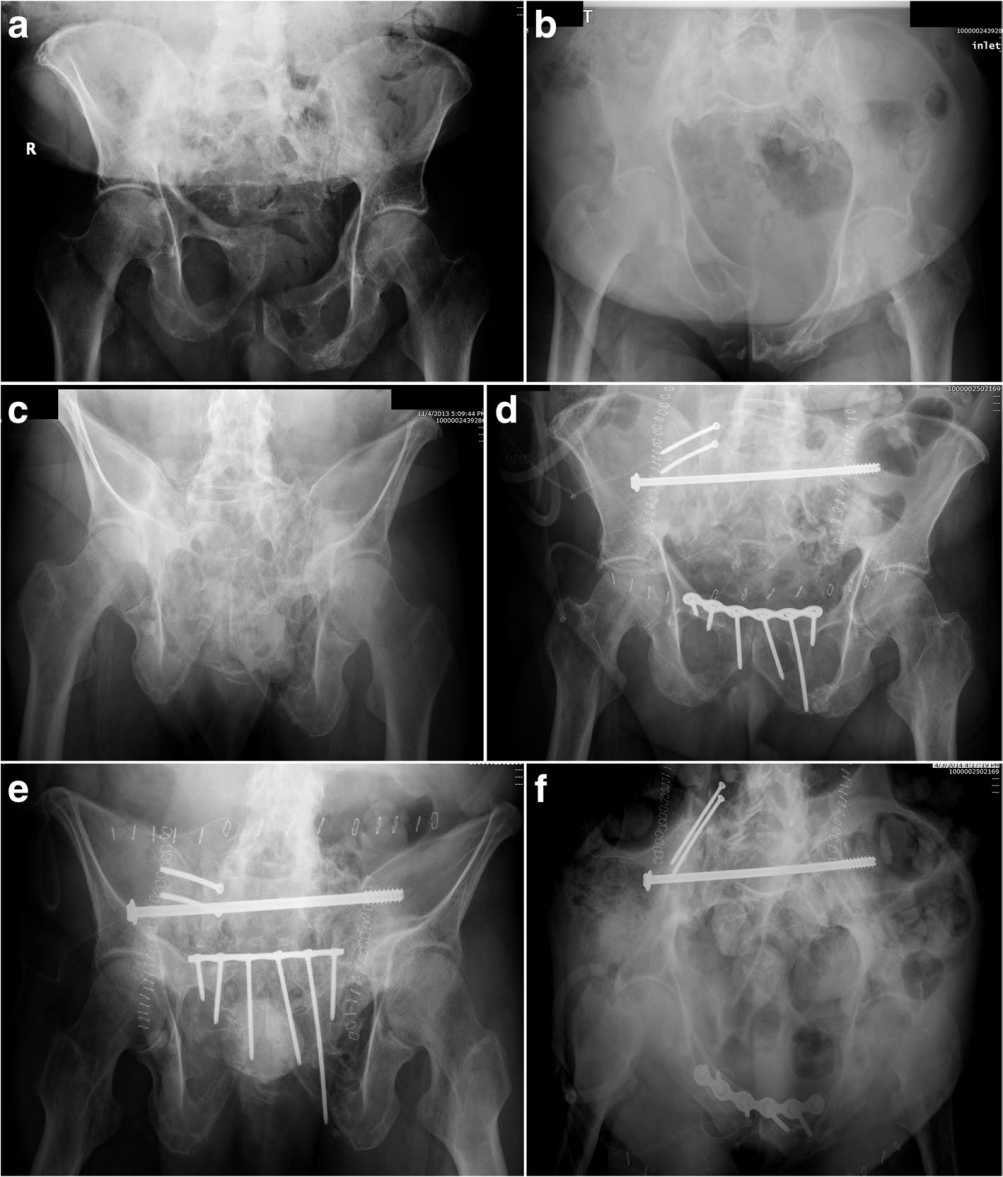
Pre- (**A**-**C**) and post-operative (**D**-**F**) radiographic images of a pelvic malunion from a right SI fracture-dislocation and pubic symphyseal dislocation. The malunion correction was performed in stages resulting in both anterior and posterior ring fixation

**Fig. 11:**
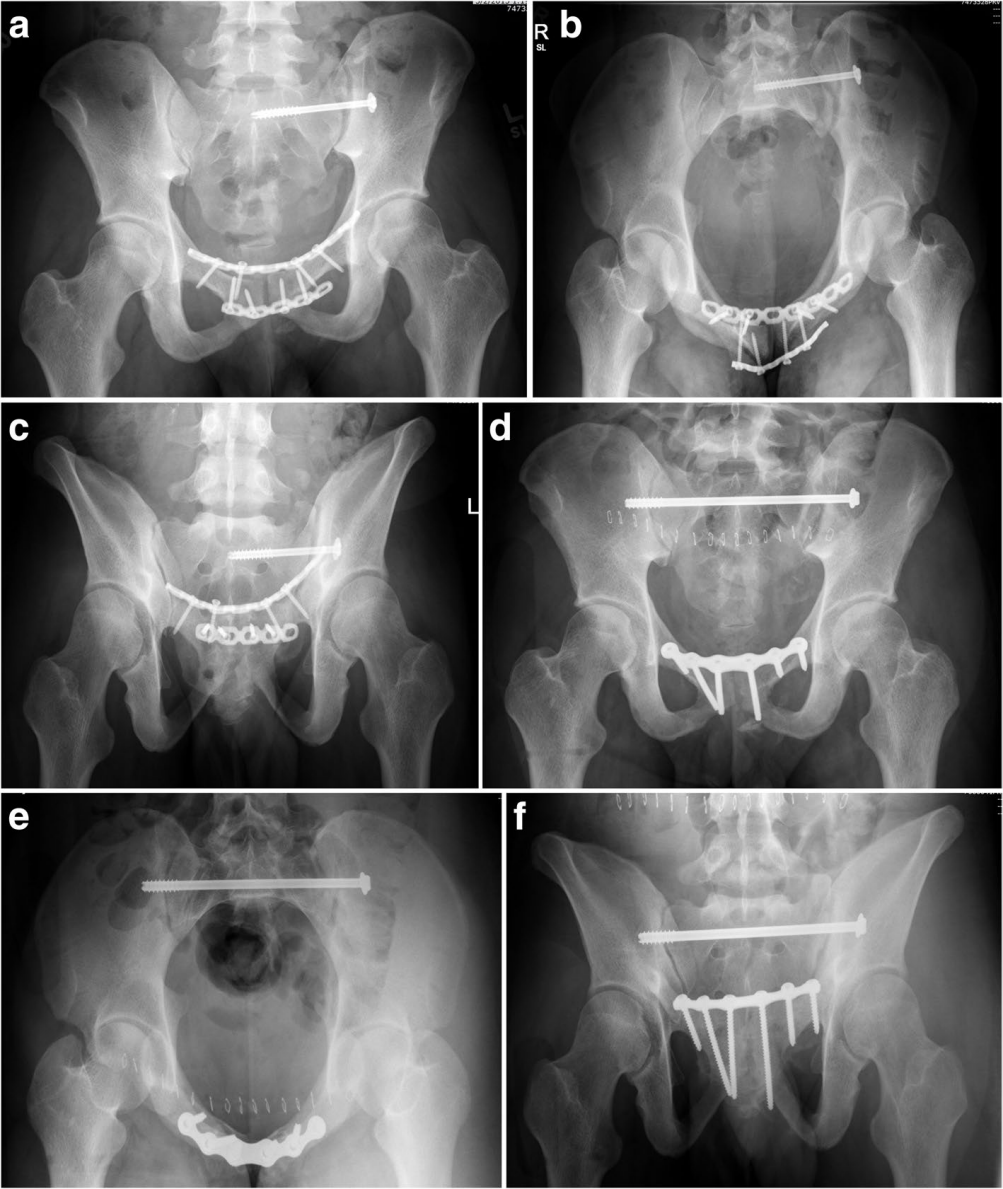
Pre- and post-operative radiographic images of a left SI fracture-dislocation and pubic symphyseal dislocation that was fixed in a mal-reduced position (**A**-**C**). The malunion was re-reduced and fixed with a trans-sacral screw and anterior ring fixation (**D**-**F**)

TSSs span across the sacrum and ideally penetrate the outer cortex of the contralateral ilium (Fig. 6a-c). As mentioned before, when a TSS is utilized for a vertical sacral fracture there is more screw in contact with the bone (both iliac cortices, SI joints, and spanning the S1 body). There is more force required to cause a rotational moment of the TSS and subsequent cranial displacement of the affected hemipelvis. Matta and Tornetta reported that longer ISS provide better fixation due to greater resistance to rotation and vertical shear stress (Fig. 7) [14]. To prevent rotation and increase fixation strength, another screw (either ISS or TSS) can be placed either in S1 or S2 (Figs. 8 and 9) [35]. We routinely utilize a washer if there is fracture displacement [36], however, surgeon discretion should be utilized to avoid over-compression of the fracture. As discussed earlier, TSSs can also be utilized in pelvic malunions/nonunions (Figs. 10 and 11).

#### Biomechanics of screw fixation

The authors routinely use a 7.3- or 8.0-mm diameter partially-threaded screw. There may be surgeon preference based on the diameter of the screw (6.5, 7.3, vs 8.0 mm) as well as partially vs. fully threaded screws. A larger screw diameter is more resistant to cantilever bending and has more mechanical advantage [37]. Mechanical advantage is defined as circumference of the screw over the pitch (*MA* = *C/P*) [37]. Thus, a larger diameter screw with a larger circumference (given equal pitch) would have a larger mechanical advantage (either in the S1 body or the contralateral iliac cortex). This mechanical advantage would confer the larger screw greater resistance to toggle in the coronal plane.

Regarding thread length, Kraemer et al. [38], in a cadaveric study, determined that long-threaded (32 mm) iliosacral screws in the sacral body had nearly 3 × the extraction strength of short-threaded screws (16 mm). Long-threaded ISS had 10 × more extraction strength than short-threaded screws placed in the sacral ala. The purchase of the distal threads of ISS or TSS is critical to compression of the SI joint/fracture and resistance to vertical shear. A screw with poor fixation in the S1 body, for example, would be more prone to toggle in the coronal plane. Therefore, when placing these screws, it is important to ensure the threads are in the S1 body and not in the contralateral ala. As mentioned before, the strongest bone is the iliac cortex, the adjoining SI joint bone, and the superior endplate of the S1 body. Thus, a longer iliosacral screw is not necessarily better; rather, it is the location of the thread purchase of the longer screw that matters. An iliosacral screw with poor thread purchase in S1 due to osteoporotic bone is more prone to toggle failure. In patients suspect for poor bone stock, it is beneficial to engage the superior endplate of S1.

Apart from the technicalities of screw insertion, the authors have not experienced any increase in patient morbidity with TSSs compared to ISSs. Other authors have also demonstrated no increase in pain or adverse outcomes when utilizing TSSs [39, 40]. The authors have a low threshold to remove either an ISS or TSS. As discussed previously, the SI joint is a mobile joint, and our patients anecdotally have less discomfort when these implants are removed.

## Conclusion

TSSs provide greater resistance to rotation and vertical shear than the ISS and may be especially useful in patients with vertical sacral fractures, pelvic malunions/nonunions, and osteopenic bone. The authors’ experiences have found TSS fixation to have lower failure rates than that of ISS fixation. Definitive treatment for posterior pelvic ring injuries is still evolving as we advance our understanding of biomechanics and fixation techniques.

### Expert recommendation

To summarize, if deemed safe, we use TSSs for the following posterior pelvic ring injuries:

1. Sacral fractures (predominantly vertically oriented fractures), young patients
2. Failed ISS fixation
3. Osteoporotic sacral fractures
4. Minimally displaced SI fracture-dislocation
5. Pelvic malunions/nonunions
6. Secondary fixation after an ISS in an unstable fracture pattern

## Data Availability

Not applicable.
